# Differential effect of histone H3.3 depletion on retroviral repression in embryonic stem cells

**DOI:** 10.1186/s13148-023-01499-5

**Published:** 2023-05-11

**Authors:** Ayellet Tal, Jose David Aguilera, Igor Bren, Carmit Strauss, Sharon Schlesinger

**Affiliations:** Department of Animal Science, The Robert H. Smith Faculty of Agriculture, Food and Environment, The Hebrew University of Jerusalem, Rehovot, Israel

**Keywords:** H3.3, Turnover, Retrovirus, Embryonic stem cells, Epigenetic silencing, Trim28

## Abstract

**Background:**

Integration of retroviruses into the host genome can impair the genomic and epigenomic integrity of the cell. As a defense mechanism, epigenetic modifications on the proviral DNA repress retroviral sequences in mouse embryonic stem cells (ESC). Here, we focus on the histone 3 variant H3.3, which is abundant in active transcription zones, as well as centromeres and heterochromatinized repeat elements, e.g., endogenous retroviruses (ERV).

**Results:**

To understand the involvement of H3.3 in the epigenetic silencing of retroviral sequences in ESC, we depleted the H3.3 genes in ESC and transduced the cells with GFP-labeled MLV pseudovirus. This led to altered retroviral repression and reduced Trim28 recruitment, which consequently led to a loss of heterochromatinization in proviral sequences. Interestingly, we show that H3.3 depletion has a differential effect depending on which of the two genes coding for H3.3, *H3f3a* or *H3f3b*, are knocked out. Depletion of *H3f3a* resulted in a transient upregulation of incoming retroviral expression and ERVs, while the depletion of *H3f3b* did not have the same effect and repression was maintained. However, the depletion of both genes resulted in a stable activation of the retroviral promoter. These findings suggest that H3.3 is important for regulating retroviral gene expression in mouse ESC and provide evidence for a distinct function of the two H3.3 genes in this regulation. Furthermore, we show that Trim28 is needed for depositing H3.3 in retroviral sequences, suggesting a functional interaction between Trim28 recruitment and H3.3 loading.

**Conclusions:**

Identifying the molecular mechanisms by which H3.3 and Trim28 interact and regulate retroviral gene expression could provide a deeper understanding of the fundamental processes involved in retroviral silencing and the general regulation of gene expression, thus providing new answers to a central question of stem cell biology.

**Supplementary Information:**

The online version contains supplementary material available at 10.1186/s13148-023-01499-5.

## Introduction

The replication of murine leukemia virus (MLV) is efficient in most dividing cells; however, it is restricted to pluripotent cells [1, 2]. After the viral DNA is integrated, the transcription of the long terminal repeat (LTR) promoter is strongly suppressed by chromatin modifiers recruited to the proviral DNA by the KRAB-ZFP binding protein, Trim28 (also known as Kap1 or Tif1b) [3]. ZFP809 recruits Trim28 into the provirus as it recognizes and binds directly to the proline primer binding site (PBSpro) sequence [4]. Trim28 recruitment leads to the involvement of several factors in the formation of heterochromatin and transcriptional silencing [5], including the H3K9 methyltransferase ESET (SETDB1), the cofactor YY1 [6], the nucleosome remodeler Smarcad1, and the heterochromatin protein HP1 [7–9]. The expression of most endogenous retrovirus (ERV) repeats is similarly restricted through a similar ensemble [6, 10, 11]. Interestingly, H3.3, Trim28, and the H3.3 chaperon DAXX were found to play a role in heterochromatin establishment on ERV sequences as the depletion of H3.3 leads to reduced H3K9me3 marking, resulting in depression of ERVs and adjacent genes [12, 13]. However, little is known about the role of H3.3 in establishing and maintaining this silencing in the MLV provirus.

A variant of Histone H3, H3.3 (coded by two genes- *H3f3a* and *H3f3b*), is a replacement histone and can be incorporated into nucleosomes in a replication-independent manner. H3.3 deposition has long been associated with gene activation, but in ESC, H3.3 is also deposited in lineage-specific bivalent genes [14] and in H3K9me3-marked endogenous retroviral sequences [15]. The two genes of H3.3 possess different 5′ and 3′ UTRs, nonidentical exon–intron, and promoter structures and are regulated by different transcriptional and post-transcriptional factors [16]. *H3f3a* and *H3f3b* also have different expression patterns in various tumors, indicating that each gene plays a different role [17]. Phylogenetic comparison and codon-usage preference analysis revealed that *H3f3b* is more ancient and evolutionarily adapted for broad expression patterns across diverse cellular programs, while *H3f3a* is more fine-tuned for a specific transcriptional program associated with cell proliferation [18]. Furthermore, the *H3f3a* mutants were viable in mice, whereas the *H3f3b* mutants were growth deficient and died at birth [19]. Together, these findings reveal the importance of H3.3 in development and differentiation and point to a partial redundancy in the function of *H3f3a* and *H3f3b*.

The dynamic deposition of H3.3 on heterochromatin and repetitive elements is performed via DAXX and ATRX [20–22]. DAXX functions as an H3.3-specific chaperon and was also shown to repress ERVs by binding the silencing complex, namely Trim28 and ESET, without the need for H3.3 chromatin deposition [23]. The enrichment of these sites coincides with the H3K9me3 marking, and the interruption of ATRX or DAXX has resulted in the loss of H3K9me3 in these regions and reduced accessibility of the ERV sequence [13]. H3.3 Deposition also depends on Smarcad1, a member of the Trim28 complex, which was suggested to carry the nucleosome eviction that allows H3.3 loading onto the retroviral genome [13]. Although retroviral sequences are enriched for H3.3, it needs to be clarified how dynamic its deposition and eviction from retroviral sequences are. While high turnover is usually correlated with DNA accessibility and active promoters and enhancers, H3.3 enrichment in repeats is related to slow turnover and closed chromatin structure. On the other hand, the chromatin accessibility of ERVs allows H3.3’s dynamic assimilation and is needed for the silencing complex binding [9, 13].

Here, we studied the incorporation levels and dynamics of H3.3 into retroviral sequences in ESCs and tested H3.3’s involvement in regulating ERV expression in these cells. H3.3 is highly enriched and hyperdynamic in the integrated MLV-like proviral sequence. Although *H3f3a* is important in the direct establishment of heterochromatin, only when both genes are depleted does repression end up being stably compromised. Furthermore, while DAXX knockout did not affect retroviral expression, Trim28 depletion activated the expression and eliminated H3.3 accumulation from proviral sequences. Our data suggest a new role for Trim28, enabling H3.3 incorporation into newly integrated viral sequences, and a role for the H3.3 genes in silencing incoming retroviruses in mouse ESCs.

## Materials and methods

### Cell culture

KH2 mouse embryonic stem cells that contain an inducible HA-tagged histone H3.3A were used (Fig. 3A) to make the study of this histone variant feasible. All ESCs were cultured in gelatinized tissue culture plates (0.2%) in standard ES cell medium (Dulbecco-modified Eagle medium (DMEM)) supplemented with 15% defined fetal bovine serum (HyClone Cat. SH30070), 100 IU/ml penicillin, 100 mg/ml streptomycin, 2 mmol/L L-glutamine (Biological Industries), 5 mg/ml non-essential amino acids, 0.12 mmol/L β-mercaptoethanol, and 1000 U/ml leukemia inhibitory factor (LIF). Furthermore, two inhibitors were added: the mitogen-activated protein kinase (MAPK)/extracellular signal-regulated kinase inhibitor (ERK) kinase (MEK) PD0325901 (1 uM) and the glycogen synthase kinase 3 (GSK3) inhibitor CHIR99021 (hereafter named 2i) at a concentration of 3uM. ESC KH2 cells were cultured under antibiotic selection with Hygromycin B at a concentration of 14 μg/ml, allowing the maintenance of a full population containing the vector containing inducible HA-H3.3A. In addition, HEK-293 T cells (Human embryonic kidney) and NIH3T3 fibroblasts (Embryonic mouse connective tissue cells) were cultured in standard tissue culture plates in Dulbecco modified Eagle medium (DMEM) supplemented with 10% defined fetal bovine serum (HyClone Cat. SH30070), 100 IU/ml penicillin, 100 mg/ml streptomycin, 2 mmol/L L-glutamine (Biological Industries). All cells were grown in a humidified incubator with a controlled environment of 5% CO2 and a temperature of 37° C. Cells were counted using TC20™ automated cell counter (Bio-Rad Laboratories Hercules). The counting was conducted by mixing 5 μL of cell resuspension with 5 μL Trypan-blue and subsequently loaded on cell counter slides (Bio-Rad #1,450,016).

### CRISPR gRNA design

The gRNA design for the genes in this research (*H3f3b, DAXX, ATRX*) was made using Zhang lab Guide Design Resources (zlab.bio/guide-design-resources). The online tools chosen are CRISPOR (crispor.tefor.net), CHOPCHOP (chopchop.cbu.uib.no) and Benchling (benchling.com), for their scoring methods and their user-friendly Ib-tools. After entering the desired gene and exon, each tool delivers a list of guides carrying different scoring, comprised of predicted efficiency (based on distance from the PAM sequence, self-complementary regions, restriction sites, G-C content, and the location of the guide within a gene), off-targets for mismatches, and repair-profile-repair-profile prediction. Each tool uses different algorithms for prediction; therefore, the highest score guides received were tested in at least one more tool for scoring. The guides which received the highest score in the second or third test were ordered and cloned into a PX-458 plasmid (Addgene plasmid # 48,138).

### Plasmid design and cloning

All plasmids were cloned by restriction/ligation cloning (standard cloning) and designed with the software Genome compiler V 2.2.88. shRNA oligos were cloned into pLKO1 vector, carrying Neomycin resistance, by digesting the plasmid with the restriction enzymes Ecor1 and AgeI (NEB #R0136 and # R0142, respectively) (# R0142, respectively). The product was run and cleaned from a 1% electrophoresis agarose gel and was ligated with an annealed shRNA oligos O/N. The plasmid was then transformed into competent DH5a E-coli bacteria, plasmid extraction by Midi-prep kit, and later sent for sanger sequencing for the cloned region (Hylabs). All sequences were > 99% complementary to the expected cloned product. *CRISPR-CAS9 plasmid-PX*-458 plasmid (Addgene plasmid #48,138). The vector was incubated with BbsI-HF® (NEB #R3539)**.** The product was run and cleaned from a 1% electrophoresis agarose gel, and ligated with an annealed sgRNAs oligo O/N. The procedure was carried out as above.

### Plasmid production

The viral synthesis was transformed into supercompetent *Escherichia coli* cells (DH5α by Invitrogen) by mixing 10 μL of the plasmid with 50 μL of supercompetent bacteria. Subsequently, cells were incubated for 20 min on ice and then, exposed to a 42° C heat shock for approximately 40 s. Later, cells were placed in recovery conditions (37° C) for 2 h, followed by centrifugation at 4000 rpm for 10 min and removal of the supernatant. Finally, transformed cells were seeded in LB-Agar, containing Ampicillin (100 μg/mL (Sigma)), and grown overnight in an incubator at 37° C. Single colonies were selected, isolated, and grown in an LB + ampicillin (10 μg/mL) overnight. Subsequently, the bacterial cells were harvested, and the plasmids were extracted from the bacteria and purified via a Purelink HiPure Plasmid Midiprep kit (Invitrogen).

### Lentiviral and retroviral packaging

#### Lentiviral particles

Replication defective lentiviral infectious particles were packaged using HEK293 T cells. Cells were seeded (2 million) in a 10 cm culture plate and grown overnight to obtain a confluent of 70–80% cells in the culture plate. Subsequently, the cells were transfected with three different plasmids containing the main components of the lentiviral particles: the plasmid containing the target shRNA sequence, an envelope-coding plasmid (pMD.G), and a plasmid coding a gag-pol sequence (psPAX2) (or for retroviral particles: the plasmid containing the target sequence (PBSpro/PBSgln GFP), a VSV-G envelope coding plasmid (pMD.G) and a plasmid coding a gag-pol sequence (pCL-ECO)) in ratio of 30:3:27 (in μg), respectively. The transfection process was facilitated using a polyethylenimine (PEI) transfection reagent (Sigma) in a DNA/PEI ratio of 1:2. Finally, the supernatant containing the lentiviral particles was harvested 48 and 72 h after transfection, filtrated through a 45 nm filter, followed by the addition of polybrene (Merck) at a concentration of 10 μg/mL to improve the infection rate.

#### Plasmid DNA transfection

ESC KH2 cells were seeded 18-24 h prior to transfection, at a density of ~ 200 K cells/Ill in a 6-well plate. On the day of transfection, the medium was removed and a mix of 250ul Opti-MEM (reduced serum medium) containing 7.5ul of Mirus TransIT®-LT1 Transfection Reagent, and 2.5ug of the cloned plasmid. Cells were incubated with the mix for 24 h and were then washed and sorted by FACSKO cell-lines creation.

KO cell lines were created by transfecting a vector (Addgene Px458) containing gRNA for either *DAXX, ATRX* or *H3f3b* (separately) and a GFP reporter, into KH2 ES cells using Mirus TransIT®-LT1 Transfection Reagent. The cells were then sorted by Fluorescence-activated cell sorting (FACS). GFP-positive cells were seeded scarcely at different concentrations (1–100 cells). Clonal isolation was made, and isolated colonies were scanned for KO by RT-qPCR, and then by Sanger sequencing in the targeted genes.

#### Viral infection

Mouse ESCs and NIH3T3 cells were plated in a six-well tissue culture plate (gelatinized in the case of ESC) in order to have approximately 200,000 cell at the day of infection. On the day of infection, growth media was removed and substituted with 750 μL of supernatant containing the viral particles, along with 2 μL LIF in the case of ESC to reduce cell differentiation. 18 to 24 h after infection, the supernatant containing the viral particles was removed and substituted with the corresponding standard growth medium for each cell line. In the case of cell KD using pLKO1 (*Neomycin R* + shRNA) lentiviral infection, the antibiotics selection started 36-48 h after infection, by supplementing the media with G418 *(12.5* μg*/*ml).

### Virus copy number determination

The difference in threshold cycle (CT) values (ΔCT) using two primers on the provirus, 40nt and PBS, was used to monitor proviral DNA copy number using RT-qPCR. Gapdh primers were used to normalize the amount of genomic DNA. A one copy number standard was established by infecting NIH3T3 cells at a very low multiplicity of infection (MOI) with the MLV-GFP vector and sorting the GFP positive cell population (< 10% of the total), ensuring that a single copy of GFP virus was present. The ratio of GFP to genomic DNA (Gapdh) in this sample is normalized to 1 (one copy of provirus per cell genome), and this sample is taken as the calibrator. The differences in ΔCTs (ΔΔCT) for the samples of interest and the calibrator are used to estimate the relative quantity (RQ) of provirus by using the formula RQ = 2-ΔΔCT. The copy number values given were obtained by averaging results from three PCR reactions. Uninfected NIH3T3 cells were used as negative control. In the flow analysis results presented in the figures, all numbers were normalized to the infection efficiency as seen by the % of NIH3T3 expressing cells. The numbers are given as GFP-positive KH2 cells/GFP-positive NIH3T3 cells × 100; this is to correct for variation in MOI when using different virus batches.

### RNA extraction, cDNA synthesis, and quantitative PCR

RNA was extracted from the cells via PureLink RNA Midikit (Invitrogen). Any DNA remnant was degraded from the product using DNase enzyme (Turbo DNase by Invitrogen, AM2238). RNA was then reverse-transcribed into cDNA using High-Capacity cDNA Reverse transcription kit (Applied Biosystems). Quantitative PCR reactions were performed using Fast SYBR Green Master Mix (Applied Biosystems) in an ABI Step-One Plus quantitative PCR system. To ensure validity, each sample was tested in triplicates (technical replicates). All primers used were tested and found to be compatible with standard curve evaluation and are shown in the primer table. The relative change in the mRNA fold was calculated with the ΔΔct method using control gene as reference.

### Flow cytometry* (*FACS*)*

Cells were detached from the plate using trypsin solution and then, filtered through a 35uM nylon filter (Thermo Fisher) into a Falcon FACS test tube (Thermo Fisher). Cell fluorescence was determined using the Cytoflex flow cytometer, equipped with a 488 nm laser. A minimum of 10,000 cells were examined for each measurement at a flow rate of 12–35 μL/second. Cell particles were excluded from the analysis using gating, and the percentage of fluorescent-positive cells was determined using the FlowJo V.10 flow cytometer software.

### Chromatin immunoprecipitation *(*ChIP*)*

For the H3.3-HA time-ChIP, 5 μg/mL of Doxycycline was added to the cell culture seven days after PBSpro/gln infection for the induction of H3.3-HA, and the cells were harvested after 1, 4 or 8 h. In other ChIP experiments, cells were plated in a 10 cm tissue culture plate two days after retroviruses infection. One day later, 5*10^6^ cells were subjected to chromatin immunoprecipitation (ChIP) after being crosslinked with 1% formaldehyde for 10 min at room temperature. The chromatin was extracted and sonicated to an average size of 300 bp. Immunoprecipitation was performed using the Magna ChIP™ kit (Millipore), and the DNA was purified using the QIAquick PCR purification kit (Qiagen). Anti-Trim28 antibody (ab22553, Abcam), anti-trimethyl-Histone H3 (Lys9) (07–442, Millipore) and anti-HA antibody (Abcam, ab9110) were used, while IgG antibody served as a negative control (sc-2027, Santa Cruz). Real-time PCR was performed for amplification, and the bound/input values were normalized using the negative control gene results as a reference (set to 1). For primer sequences, see Additional file 2: Table S1. Turnover rates were estimated by taking the log2 of the ratio between the 1 h and 8 h H3.3-HA% input ChIP. This ratio allows us to estimate the overall dynamics by which each proviral or genomic locus accumulates newly generated H3.3 nucleosomes. At the first time points (e.g., 1 h), only very dynamic loci show the incorporation of H3.3 into the nucleosomes, resulting in strong ChIP-seq signal for these few positions. Thus, the ratio between early (1 h) and late (8 h) time points offers a direct approximation of turnover dynamics. We take the logarithm of this ratio (in base 2) to allow more accurate visualizations.

### Immunofluorescence

To confirm the presence of the Ha-Tag in the Kh2 ESC, 50,000 cells were grown in gelatinized microscope coverslips inside a 12 well tissue culture plate supplemented with standard ESC cells media described above. Subsequently, synthesis of H3.3-Ha was induced in cells for 1, 4 and 8 h by adding 5 μg/ml of Doxycycline (Dox) into cell culture media. Next, cells were washed twice with PBS and fixated with 300 μL paraformaldehyde per well and incubated for 15 min at room temperature. After fixation, cells were washed three times with PBS and blocked with PBS + 20% FBS for 1 H. Once cell blocking was completed, cells were incubated at 4° C overnight with PBS + 20% FBS and ChIP grade anti-HA tag antibody (ab9110, Abcam) in a 1:300 ratios. The next day, cells were washed three times with PBS for 5 min and then, incubated with secondary goat anti-mouse IgG (H + L) antibody, FITC (Thermo Fisher, F-2761) in a 1:1000 ratio on PBS + 20% FBS. Subsequently, cells were washed three times with PBS for 5 min and later incubated with 300 μL PBS with 5 μg/mL DAPI Fluoromount-G (SouthernBiotech, 0100–20) for 15 min. Afterward, cells were washed one last time with PBS and then, mounted into a microscope slide using VECTASHIELD Antifade Mounting Medium (Vector Laboratories H-1000).

### Co-immunoprecipitation

For whole cell extraction, trypsinized cells were washed with PBS and resuspended in ice-cold hypotonic lysis buffer (20 mM Tris–HCl pH 7.5, 0.2 mM ethylenediaminetetraacetic acid (EDTA), 0.5 mM 1, 4-dithiothreitol (DTT), 1 mM1 mM NaVO4, protease inhibitor 1:100 [Sigma]). Cells were incubated for 10 min on ice. A similar volume of high salt buffer (20 mM Tris–Cl pH 7.5, 0.2 mM EDTA, 0.5 mM DTT, 1 M NaCl) was added, and cells were incubated 15 min on ice. Cells were then centrifuged (20,000 × *g*, 4 °C, 30 min), and the supernatants were collected for further analysis. The extracts were diluted with immunoprecipitation wash buffer (IP) (20 mM Tris–HCl pH 7.5, 0.2 mM EDTA, 0.5 mM DTT, 0.2% Triton X-100, 150 mM NaCl) to a final volume of 400 µL. 4 μg of antibody was added to the lysate and incubated overnight in 4 °C, with rotation. The beads (Dynabeads; Invitrogen) were magnetically pulled as recommended by the manufacturer and washed 3 times with IP wash buffer. After the final wash, beads were incubated with 2 × SDS loading buffer, heated to 95 °C for 3 min, and the supernatant was collected and observed by Western blot. The antibodies used for pulldown and Western blot were anti-Trim28 (ab22553, Abcam), anti-IgG (SC-69916, Santa Cruz), and anti-GAPDH (25,778, Santa Cruz). Detection for Western blot was with HRP conjugated anti-mouse or anti-rabbit antibodies (115–035-062, 111–035-144, respectively, Jackson ImmunoResearch, Pennsylvania, USA). The quantification of the WB band intensity was done using ImageJ, and the values shown are normalized to the WT sample.

## Results

### Dynamic H3.3 incorporation onto retroviral sequences

**Fig. 1:**
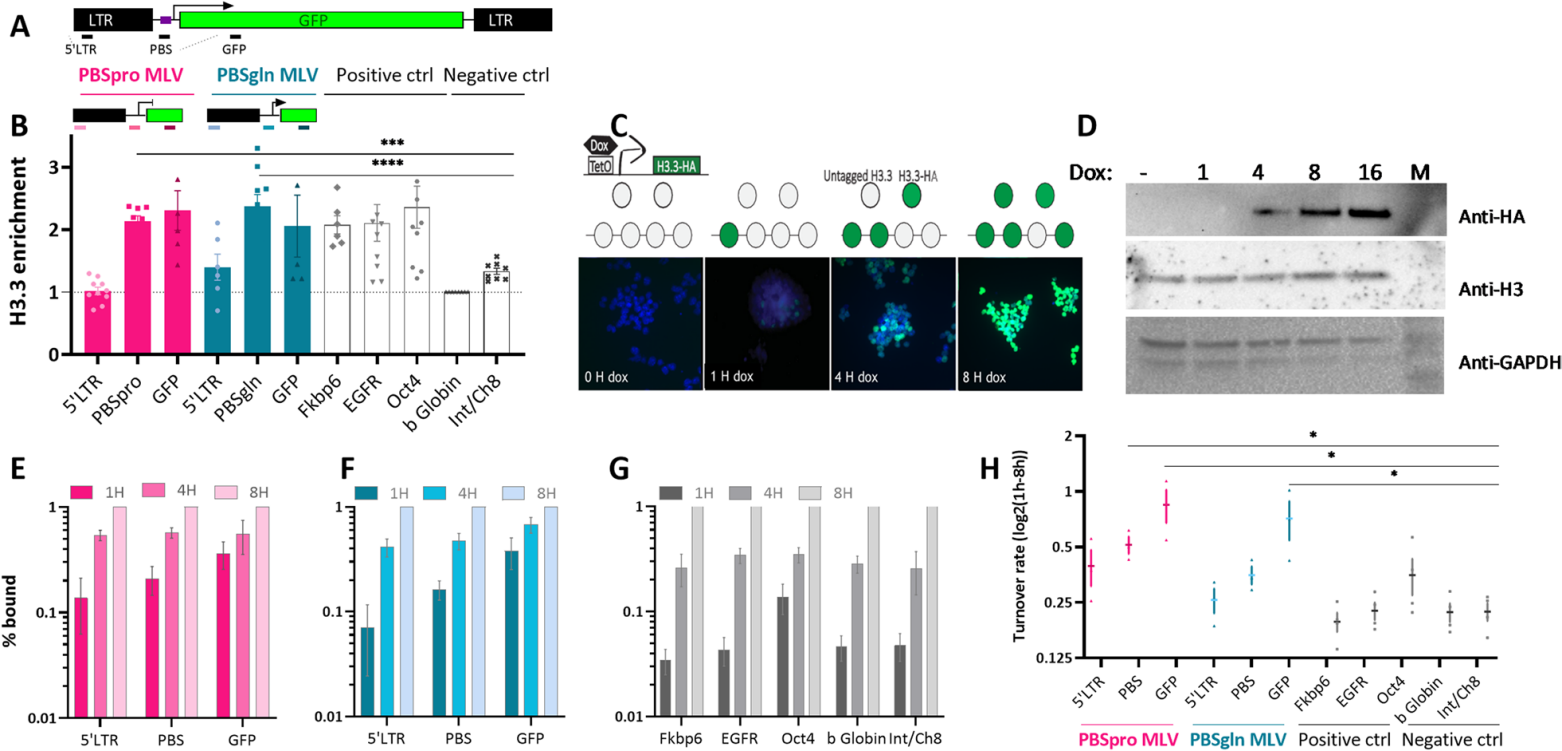
Measuring of H3.3 deposition and dynamics on the MLV provirus. **A** Illustrating the retroviral insert with the PBSpro and PBSgln variation, the primers used are marked by pink or blue lines, respectively. **B** Anti-HA ChIP on MLV-infected, Dox-induced KH2 cells followed by RT-qPCR on proviral and genomic targets. Values are % Input normalized to the b-Globin gene. Data are the mean ± s.e.m. (*n* = 4). **C** A schematic diagram showing the doxycycline (Dox) histone H3.3-HA induction system and the use of this system for assaying H3.3 turnover dynamics. HA-tagged H3.3 expression was induced by Dox addition at different times and immunolabeled with anti-HA antibodies. DAPI staining overlay is shown in blue (**D**) immunoblotting of Dox-induced KH2 cells to extract for the indicated hours using anti-HA, with anti-H3 and GAPDH as loading controls. **E** Accumulation of H3.3-HA into PBSpro, **F** PBSgln and **G** genomic sequences at 1, 4 and 8 h after DOX induction. Presented are Bound values normalized to the 8 h Bound fraction. ChIP-qPCR data are the mean ± s.e.m. (*n* = 3 for PBSpro and PBSgln, *n* = 4 for the genomic sequences). **H** Turnover rate of the proviral and genomic sequences was measured using log2 of %Input ChIP data for 1H divided by 8H. Data are the mean ± s.e.m. (*n* = 3–4). All *P* values were calculated using two-tailed unpaired Mann–Whitney U test, *P < 0.05, ***P < 0.001, ****P < 0.0001

To demonstrate the involvement of H3.3 in retroviral silencing, we used a mouse ESC line (KH2) that carries a single copy of tagged histone H3.3A located downstream of the Type I Collagen (Col1A1) locus, under the control of Doxycycline (DOX, as in [24]). A pool of the HA-tagged H3.3A accumulates immediately after Dox addition and reaches saturation after about eight hours. The cells were infected with a retroviral vector in which the viral genes were replaced with the GFP reporter gene [25]. Two vectors are used in all experiments: one with the retroviral sequence PBSpro—the tightly repressed WT MLV sequence—and the other in which the WT proline sequence was replaced with glutamine (PBSgln)—and the silencing is compromised due to a deficiency in ZFP809 binding [4] (as illustrated in Fig. 1A). To measure the deposition of H3.3-HA in PBSpro vs. PBSgln, chromatin immunoprecipitation (ChIP) was performed seven days after retroviral infection and 8 h after DOX addition. The anti-HA-ChIP data revealed enrichment on both viral vectors (Fig. 1B), suggesting that the accumulation of H3.3 on retroviral sequences is not dependent on KRAB-ZFP809 binding. Notably, the enrichment is observed on the PBS sequence and downstream, but not in the 5'LTR. The enrichment is specific to integrated proviral sequences as integrase-deficient (IND184A, a kind gift from Prof. SP Goff) PBSpro MLV showed lower H3.3 enrichment (Additional file 1: Figure S1A). To determine the H3.3 turnover rate on proviral sequences, we performed time-ChIP (as described in [24]) on cells infected with PBSpro and PBSgln after incubation with doxycycline for 1 h, 4 h, and 8 h (Fig. 1C). Immunoblotting was performed to verify that the expression levels of the variant are similar to the cellular H3 levels (Fig. 1D). To calculate the H3.3 turnover rate, we establish an 8 h enrichment level as the maximum enrichment possible. Next, we utilized the enrichment levels in addition to the addition of 1H doxycycline and normalized them to the 8H enrichment levels, and then, the data were transformed to a logarithmic scale. As a result, we obtained the incorporation ratio for each time point compared to the maximum enrichment. Both proviral sequences of PBSpro (Fig. 1E) and PBSgln (Fig. 1F) showed high relative enrichment (> 0.1 Bound) already after one hour, suggesting fast incorporation of newly synthesized H3.3-HA into proviral chromatin. Looking at the genome, only Oct4 showed similar fast dynamics, while the other two highly H3.3 enriched genes presented slow incorporation dynamics (Fig. 1G). The effect was H3.3 specific since the ChIP control H3 antibody did not show differences over time, as expected (Additional file 1: Figure S1B). This, together with previous findings [24, 26–28], shows that the levels of H3.3 enrichment are not necessarily correlated with the turnover rate. Calculating the turnover rate as previously described (log2 (1 h/8 h HA enrichment), as in [24]) suggests a fast and dynamic exchange of H3.3 on proviral sequences (Fig. 1H). In addition, the turnover rate of the highly repressed PBSpro was slightly quicker than that of the more transcriptionally active PBSgln provirus, and both faster than that of the genomic highly expressed gene (e.g., Oct4). Therefore, dynamic nucleosome repositioning is not necessarily correlated with either expression or H3.3 enrichment. Together, these data show that H3.3 is enriched in proviral sequences and that the accumulation of this variant in the viral sequences is hyperdynamic.

### The retroviral repression is impaired in H3.3-depleted cell lines

**Fig. 2:**
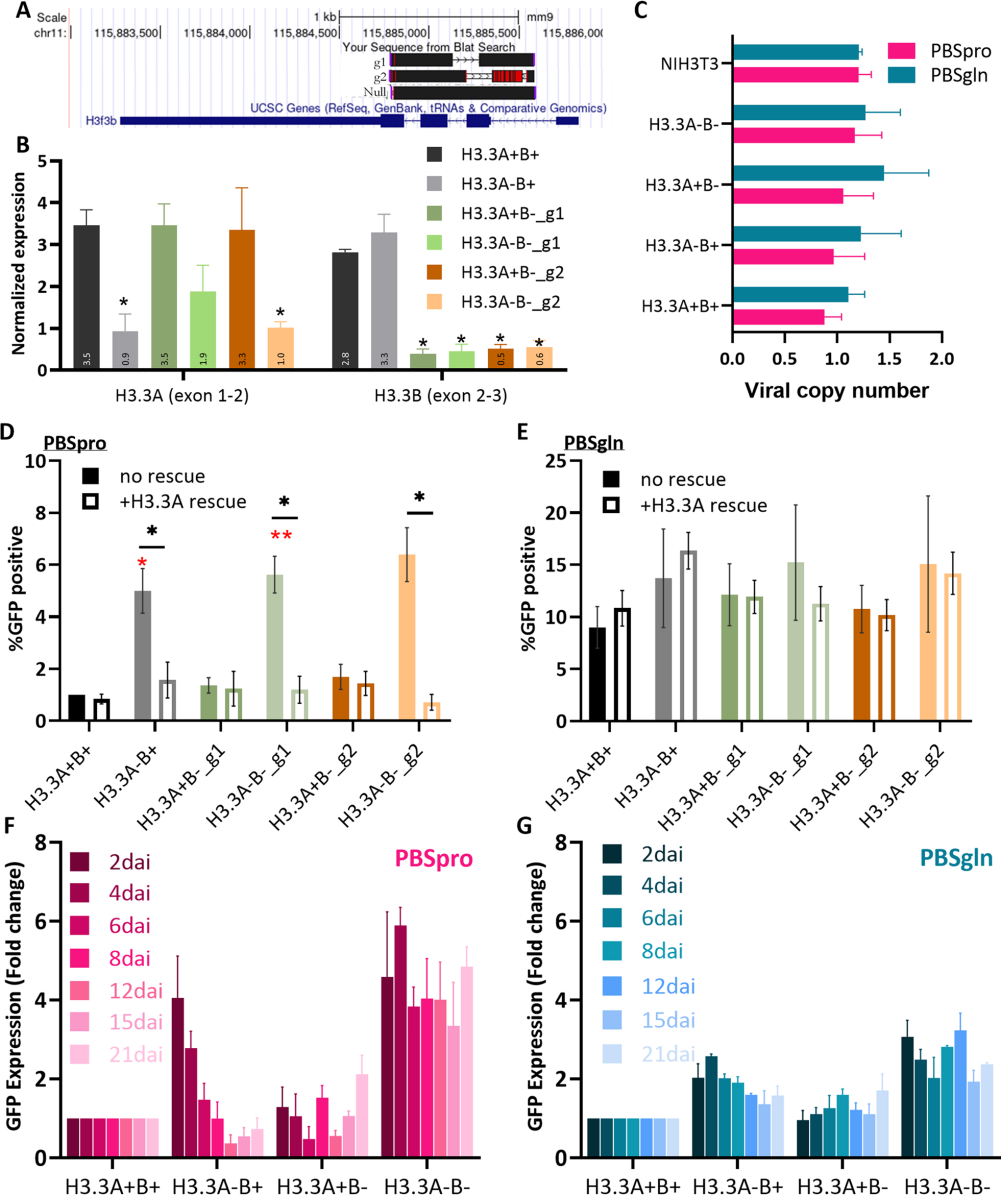
Effect of H3.3 depletion on proviral expression. **A** Sanger sequencing of the first coding exon of *H3f3b* gene following Crispr/Cas9 transfection and sorting of clones. **B** Following infection with lentiviral vector caring antisense H3f3a shRNA, full depletion of H3.3 was verified by RT-qPCR, normalized to UBC control gene. Data are the mean ± s.e.m. (*n* = 3) **C** Viral copy number calibration of PBSpro/PBSgln MLV pseudovirus. Levels of viral insertion into the cell genome were determined and normalized to NIH3T3 cells carrying a single integration site. **D** The percentage of GFP-positive after infection by PBSpro or **E** PBSgln virus in the H3.3 depleted ESCs, normalized to the WT PBSpro GFP expression. The red asterisk shows a significant difference from WT ESC (One sample Wilcoxon test), and the difference between the cells before and after the restoration of H3.3 expression is denoted by a black asterisk. Data are the mean ± s.e.m. (*n* = 5) **F** GFP expression levels of H3.3 depleted ESC, infected with PBSpro or **G** PBSgln MLV virus, days after infection (dai). All *P* values are calculated using a two-tailed unpaired Mann–Whitney U test; Data are the mean ± s.e.m.*P < 0.05, **P < 0.01

To examine whether H3.3 accumulation in the proviral sequence is linked with the retroviral repression in ESC, we depleted the expression of the two histone H3.3 genes (*H3f3a* and *H3f3b*) that translate into identical protein products (H3.3A and H3.3B) [29]. First, to knockout H3.3B, we used two designed gRNA targeting the second exon of the *H3f3b* gene and obtained two CRISPR KO lines (g1- and g2-H3.3B-). KO was verified using Sanger sequencing (Fig. 2A) and RT-qPCR (Fig. 2B). Next, to deplete H3.3A, wild type (WT) and H3.3B- cells were infected with a pLKO1 lentiviral vector carrying shRNA that targets the *H3f3a* gene (KD). Depletion was verified using RT-qPCR (Fig. 2B). The four cell lines (H3.3A + B + , H3.3A-B + , H3.3A + B-, H3.3A-B-) were infected using PBSpro or PBSgln MLV vectors. The depletion of H3.3 did not affect the efficiency of retroviral integration (Fig. 2C). The percentage of cells expressing GFP was measured by flow cytometry and normalized to the GFP levels of differentiated mouse fibroblasts (NIH3T3 cells). Interestingly, the expression of GFP of the PBSpro virus was significantly increased in H3.3A-B + and H3.3A-B- cells (Fig. 2D, no rescue). Induced H3.3A ectopic expression by Dox addition rescued the viral repression back to the WT levels in all cell types, indicating that H3.3 plays a role in silencing incoming retroviruses in ESC (Fig. 2D, + H3.3A rescue). This effect was not visible in the PBSgln-infected cells (Fig. 2E). This result was reproducible over independent experiments using different knockdown clones and required depletion of H3.3A, while the sole KO of H3.3B had almost no effect on the viral expression. To exclude the possibility that the method of depletion is the cause of the difference between the genes (although the significant change in viral repression was observed in the shRNA depleted cells, which still express 10–20% of the WT H3.3A levels, and not in the KO line that does not express H3.3B at all). We used shRNA to deplete H3f3b from the WT KH2 cells.

However, the GFP levels proved similar in both depletion methods (Additional file 1: Figure S1C). We further investigated the kinetics of viral expression by recording GFP expression every 2–4 days after infection (2d, 4d, 6d, 8d, 12d, 15d, 21d). Remarkably, the silencing was restored in the H3.3A-B + cells but not in the H3.3A-B- cells (Fig. 2F), suggesting slow compensation of H3.3B in the absence of H3.3A. Again, the phenotype has not changed significantly in the PBSgln-infected cells (Fig. 2G). Together, these data indicate that H3.3 deposition on retroviral sequences plays a role in their transcriptional silencing in ESCs.

### H3.3 depletion results in a reduced recruitment of Trim28 to the viral PBS region and loss of heterochromatinization at proviral sequences.

**Fig. 3:**
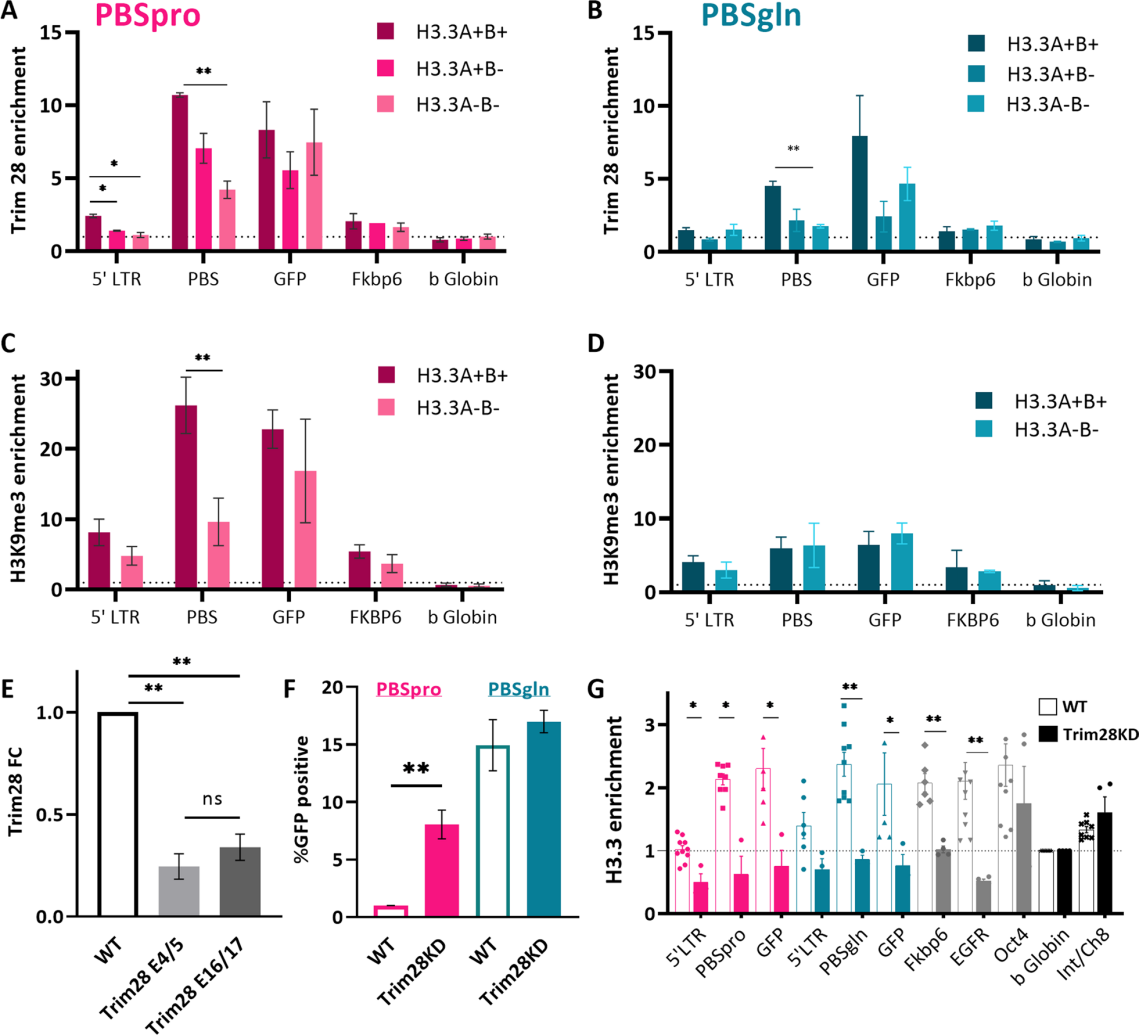
Altered chromatin conformation on proviral chromatin following H3.3 and Trim28 depletion. **A** ChIP-qPCR for Trim28 in WT and H3.3 depleted cells infected with PBSpro or **B** PBSgln virus. Data are the mean ± s.e.m. (*n* = 3) **C** anti-H3K9me3 ChIP-qPCR in WT and H3.3 depleted cells infected with PBSpro or **D** PBSgln virus. In **A**–**D**, Data are the mean ± s.e.m. (*n* = 3), *P* value was calculated using Bonferroni–Dunn method without assuming a consistent SD **E** RT-qPCR for Trim28 expression on WT and Trim28KD cells, normalized to UBC control gene (*n* = 6). *P* value was calculated using Kruskal–Wallis nonparametric test with Dunns' multiple comparison, **P < 0.01. **F** Percentage of GFP expressing cells following Trim28 depletion (*n* = 6). **G** ChIP-qPCR for H3.3-HA in WT and Trim28KD ESCs infected with PBSpro or PBSgln viral vectors (*n* = 2). In **F**, **G**, Data are the mean ± s.e.m., *P* value was calculated using Mann–Whitney U unpaired nonparametric test, *P < 0.05, **P < 0.01

To assess the chromatin landscape at the proviral sequences in ESCs, we performed chromatin immunoprecipitation using antibodies recognizing Trim28 and H3K9me3, followed by qPCR analysis (ChIP-qPCR) of selected genomic regions and retroviral sequences in control and H3.3-depleted ESCs. Trim28 recruitment to the PBSpro sequence by ZFP809 [4] is crucial for retroviral silencing in ESC [3, 30]. In the absence of H3.3, there was a distinct reduction in Trim28 enrichment from the viral PBS region (Fig. 3A, B). Trim28 is known to recruit SETDB1-mediated trimethylation of H3K9 (H3K9me3), which plays a pivotal role in silencing endogenous and exogenous retroviral sequences [6]. Subsequently, a decrease in H3K9 trimethylation was also observed from the PBSpro (Fig. 3C) but not in the PBSgln (Fig. 3D). Notably, we observed no difference in H3K9me3 enrichment in silenced genes such as FKBP6 and b-Globin in the absence of H3.3, yet H3.3 depletion had a considerable effect on Trim28 deposition and H3K9me3 levels in the proviral sequence. To determine whether H3.3 enrichment was reduced in the absence of Trim28, we used shRNA targeting Trim28 as in [25] and examined the depletion of both Trim28 splicing variant by RT-qPCR (Fig. 3E). Using co-immunoprecipitation with HA antibody, we showed interaction of H3.3 with Trim28 in WT cells (Additional file 1: Figure S1D). As expected, Trim28 depletion caused a significant increase in viral expression (Fig. 3F); however, H3.3 accumulation was omitted from the retroviral sequences (Fig. 3G). Comparison of enrichment at the positive control gene FKBP6 suggests that this omission might not be limited to retroviral sequences. Reduced H3.3 enrichment cannot be attributed to reduced nucleosome occupancy upon depletion of Trim28, as overall levels of H3 show little variation (Additional file 1: Figure S1E).

### The interaction between Trim28 and H3.3 is not bridged by DAXX.

**Fig. 4:**
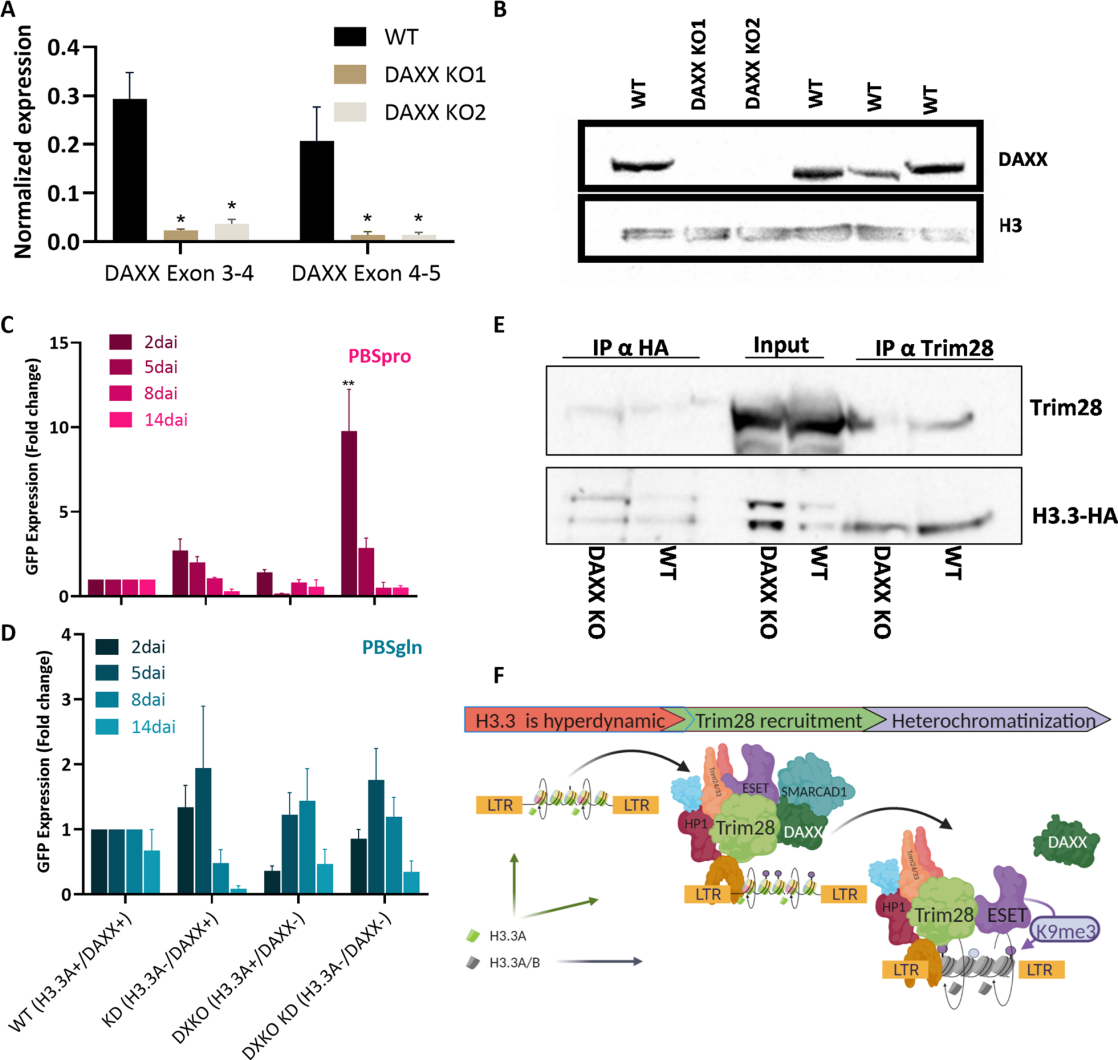
The interaction between Trim28 and H3.3 is not mediated by DAXX. **A** RT-qPCR for DAXX expression on WT and DAXX KO cells, normalized to UBC control gene. Data are the mean ± s.e.m. (*n* = 4) *P* value was calculated using Mann–Whitney U unpaired nonparametric test, *P < 0.05. **B** immunoblotting WT and DAXX KO cells using anti-DAXX and H3 antibody as a control. **C** Percentage of GFP expressing cells following DAXX depletion. Cells infected with PBSpro or **D** PBSgln virus. Data are the mean ± s.e.m. (*n* = 3) *P* value was calculated using Kruskal–Wallis nonparametric test with Dunns' multiple comparison, **P < 0.01 **E** Immunoprecipitation (IP) of nuclear extracts from WT or DAXX KO ESCs carrying the H3.3-HA-tag, followed by immunoblotting with the indicated Antibodies. **F** Suggested model describing three phases of silencing onset: (1) H3.3 hyper dynamic nucleosome deposition is required for proper (2) Trim28 recruitment to the proviral DNA, which then (3) facilitates heterochromatinization of the region. At this point, DAXX is not required for H3.3 accumulation. Created with BioRender.com

To pinpoint the factors that recruit H3.3 to retroviral sequences, we eliminated one of the specific chaperones of H3.3, DAXX. The protein was depleted using gRNA targeting the third exon of the gene. KO was confirmed by RT-qPCR (Fig. 4A) and immunoblot (Fig. 4B). DAXX depletion did not affect PBSpro and PBSgln transcription, but when combined with H3.3A KD, a significant up-regulation of PBSpro expression was observed (Fig. 4C). The results show that the silencing is restored after a week or so, probably as H3.3B still exists in the system and can therefore compensate for H3.3A loss. When cells were infected with the PBSgln virus, DAXX KO did not affect viral transcription, in agreement with the lack of effect observed in H3.3-depleted cells (Fig. 4D). Next, co-IP in cells for Trim28 and H3.3 shows that Trim28 still binds to H3.3 even in the absence of DAXX (Fig. 4E). This could point to another mechanism involved in the incorporation of H3.3 into repressed regions and explain the little effect of DAXX KO on the retroviral transcriptional regulation pattern.

## Discussion

The effort to decipher the molecular mechanism of retroviral silencing in pluripotent cells has been ongoing for decades; however, many key questions remain [31]. Here, we examined the exchange rate of histone variant H3.3, a critical regulator of dynamic chromatin, in retroviral sequences in pluripotent cells and investigated the role of H3.3 in regulating retroviral transcription in these cells. Our results show that H3.3 is highly enriched and hyperdynamic in integrated MLV-like proviral sequences in pluripotent cells. We also found that H3.3 depletion leads to impaired retroviral repression and reduced recruitment of Trim28 to the viral PBS region, resulting in a loss of heterochromatinization in proviral sequences. Our findings suggest that H3.3 plays a crucial role in regulating retroviral gene expression and that its incorporation into retroviral sequences may be important in maintaining retroviral silencing (Fig. 4F). Based on the difference in the depletion of the effect of the two H3.3 genes, we hypothesize a mechanism in which H3.3A is a crucial element for the immediate onset of retroviral repression. The loss of H3.3A initiates an elevation in exogenous and endogenous retroviral elements that could be restored only after five days by the functional H3.3B protein.

### The dynamic incorporation of H3.3 onto retroviral sequences

The different mechanisms underlying histone deposition and eviction (that is, turnover) relate to the dynamics of H3.3 at different regions of the chromatin [26]. In ESCs, a high abundance of H3.3 is not restricted to active genes but is also found around promoters of silent genes and repressed repetitive elements [12, 15, 32], suggesting that the histone variant H3.3 is involved in ERV regulation. However, the dynamic incorporation of H3.3 onto the incoming, strictly silenced proviral MLV sequence was never examined. Using our Time-ChIP method, we validated that high turnover correlates with DNA accessibility of active genomic sites (Fig. 1G, H and [33]). However, when comparing the H3.3 dynamics of strictly repressed MLV PBSpro with that of the more expressed PBSgln, we observe an unusually high H3.3 turnover (which we called “hyperdynamic”) in both, and the more transcriptionally silent PBSpro is also more dynamic. This counterintuitive correlation can be explained by the link between Trim28 mediated nucleosome eviction and H3.3 loading into the retroviral genome [13].

Therefore, a model can be suggested in which the efficient recruitment of Trim28, mediated by the PBSpro sequence of the MLV genome, is responsible for the hyperdynamic H3.3 turnover on these sequences. Additionally, it should be noted that our experimental system is highly attuned to the new H3.3 variants, while the repositioning of old H3.3 is invisible to us. This is because the incoming proviral sequences do not have any old H3.3 to recycle; therefore, only new H3.3 can be acquired on their chromatin. Furthermore, the one-hour DOX induction labels only newly transcribed H3.3, while the old ones are only observed after ~ 8 h. Together, these might explain the hyperdynamic turnover rate in integrated proviruses and its link to heterochromatinization, since the old H3.3 are more enriched for active PTMs [34, 35].

### The role of H3.3A and B in retroviral repression

Despite the high correlation between H3.3 dynamic deposition and Trim28 recruitment to retroviral sequences, its cause remains unknown. Therefore, we deleted or depleted the two H3.3 genes and examined the effect on MLV repression onset and maintenance. The results show that H3.3A depletion leads to short-term up-regulation of retroviral-derived transcription, while H3.3B deletion does not have such an effect. However, the effect of H3.3A is limited, as silencing is restored after two weeks due to H3.3B expression. This, with the consistent expression upregulation observed when both genes were eliminated, highlights the importance of H3.3 incorporation into the silenced retroviral chromatin.

Although H3.3A and B are identical on the protein level, *H3f3a* and *H3f3b* appear regulated by different transcriptional and post-transcriptional factors, such as microRNAs [16] and *H3f3b* also have distinct expression patterns in various tumors [17, 36]), and KO mice show different phenotypes [19, 37]. This, together with codon usage and genomic analysis, indicates that *H3f3b* is evolutionarily adapted for broad expression patterns in diverse cellular programs, including cell differentiation, while *H3f3a* is more fine-tuned for a specific transcriptional program [18]. Consequently, we find that H3.3A is fine-tuned for retroviral silencing, while H3.3B is less essential. Future experiments should examine the role of the noncoding regions of those two genes in regulating heterochromatin in general, and of H3.3 exchange in retroviral sequences specifically.

### The impact of H3.3 depletion on Trim28 recruitment to proviral sequences.

Our data demonstrated that depletion of Trim28 results in reduced H3.3 occupancy on retroviral sequences, and that depletion of H3.3 leads to less Trim28 enrichment. Hence, the mutual dependence between Trim28 and H3.3 controls the epigenetic silencing in these regions. Trim28 was previously shown to recruit ESET and DAXX-ATRX, which modify the H3K9me3 mark and maintain H3.3 on the chromatin, respectively. Therefore, the effect of Trim28 KD on the occupancy of the retroviral sequences with H3.3 (Fig. 3H) was expected: in Trim28 KD cells, evicted nucleosomes cannot be replaced. Interestingly, H3.3 depletion also affects Trim28 recruitment to the PBS site of the provirus, although to a lesser extent (Fig. 3A). This aligns with the idea that H3.3 stabilizes Trim28’s deposition in the silencing complex [38]. Together, these findings suggest a novel role for H3.3 in regulating retroviral elements, along with Trim28 [12]. Could H3.3 dynamic deposition onto the PBS region assist in anchoring the Trim28 protein to the proviral sequences?

### The role of DAXX in the interaction between H3.3 and Trim28

Previous studies have shown that the chaperone complex composed of ATRX and DAXX is responsible for the deposition of H3.3 on heterochromatin throughout the genome of mouse ESCs [12, 20, 21, 38, 39]. DAXX has been suggested to repress ERV by binding the silencing complex, Trim28 and ESET, without needing H3.3 chromatin deposition [12, 23, 40]. However, the participation of Trim28 in the recruitment of DAXX and H3.3 to ERVs is still a topic of debate as the enrichment of DAXX and H3.3 at these sites coincides with H3K9me3, [13, 41]. In this study, we examine the role of DAXX in the H3.3 dependent repression of the MLV provirus. Our results show that while the immediate onset of silencing depends on both H3.3A and DAXX, the slower silencing by H3.3B does not require DAXX (Fig. 4C). Furthermore, co-IP showed that Trim28 could still bind to H3.3 even without the presence of DAXX. From this, we infer that DAXX is not essential for H3.3 incorporation, or the integrity of the silencing complex. It is likely that upon depletion of DAXX, different complex deposits H3.3A and helps facilitate the repression. We hypothesize a scenario in which HIRA compensates for the loss of DAXX and holds the capacity to aid the repression complex by binding Trim28, but more research is needed.

H3.3 Histone variant deposition plays a key role in endogenous retroviral regulation in ESCs [12, 38, 42, 43]. However, the role of H3.3 in regulating incoming retroviral sequences has not yet been elucidated. As shown here, hyperdynamic incorporation into incoming retroviral sequences is essential for epigenetic retroviral silencing in mouse pluripotent stem cells. If H3.3 is depleted, Trim28 enrichment levels in proviral sequences drop significantly, leading to low H3K9me3 levels and the induction of retroviral expression. Taken together, our results emphasize the importance of H3.3 deposition for retroviral silencing and suggest a surprising distinction between the two H3.3 genes. Although coding for identical proteins, the H3.3A and B genes differ in their genetic information. This causes a different effect on retroviral silencing. It is also surprising that while the immediate onset of silencing depends on both H3.3A and DAXX, the slower silencing by H3.3B does not require DAXX. In the future, it would be interesting to examine the impact of H3.3 on retroviral silencing after differentiation onset and determine the role of other factors such as ESET and HERA in H3.3 deposition on these sequences.

## Conclusion

We identified a role for H3.3 in establishing and maintaining the repression of newly integrated retroviral sequences in ESCs. This silencing is epigenetic and the loss of H3.3, mainly H3.3A, up-regulates retroviral expression via the erasure of H3K9me3. This can serve as a paradigm for understanding the unique properties of the “retroheterochromatin” in mESC.

## Supplementary Information

**Additional file 1: Figure S1.** Controlling H3.3 deposition and dynamics.anti-HA ChIP on full length or integration deficient MLV-infected, Dox-induced KH2 cells followed by RT-qPCR on proviral site. Values are % Input normalized to the b-Globin gene. Data are the mean ± s.e.m..Accumulation of H3 into PBSpro, PBSgln, and genomic sequences at 1, 4 and 8 h after DOX induction. Presented are bound values normalized to the 8 h Bound fraction. ChIP-qPCR data are the mean ± s.e.m..Comparable levels of GFP positive cells in H3.3B KOand KDinfected with PBSpro or PBSgln MLVImmunoprecipitationof nuclear extracts from ESCs carrying the H3.3-HA-tag, followed by immunoblotting with the indicated Antibodies.ChIP-qPCR for H3 in cells depleted with WT and Trim28 infected with PBSpro or PBSgln virus. Data are the mean ± s.e.m..

**Additional file 2: Table S1.** Primers list.

## Data Availability

Not applicable.

## Abbreviations

H3.3: Histone 3.3
MLV: Murine leukemia virus
ESC: Embryonic stem cells
ERV: Endogenous retroviruses
PBSpro/gln: Proline/glutamine primer binding site
DOX: Doxycycline
RT: Reverse transcription
ChIP: Chromatin immunoprecipitation
qPCR: Quantitative polymerase chain reaction
