# Supplementary figures and images for: Differential effect of histone H3.3 depletion on retroviral repression in embryonic stem cells
Source: Clin Epigenetics. 2023 May 11;15:83. doi: 10.1186/s13148-023-01499-5 (PMC10176700; doi:10.1186/s13148-023-01499-5)

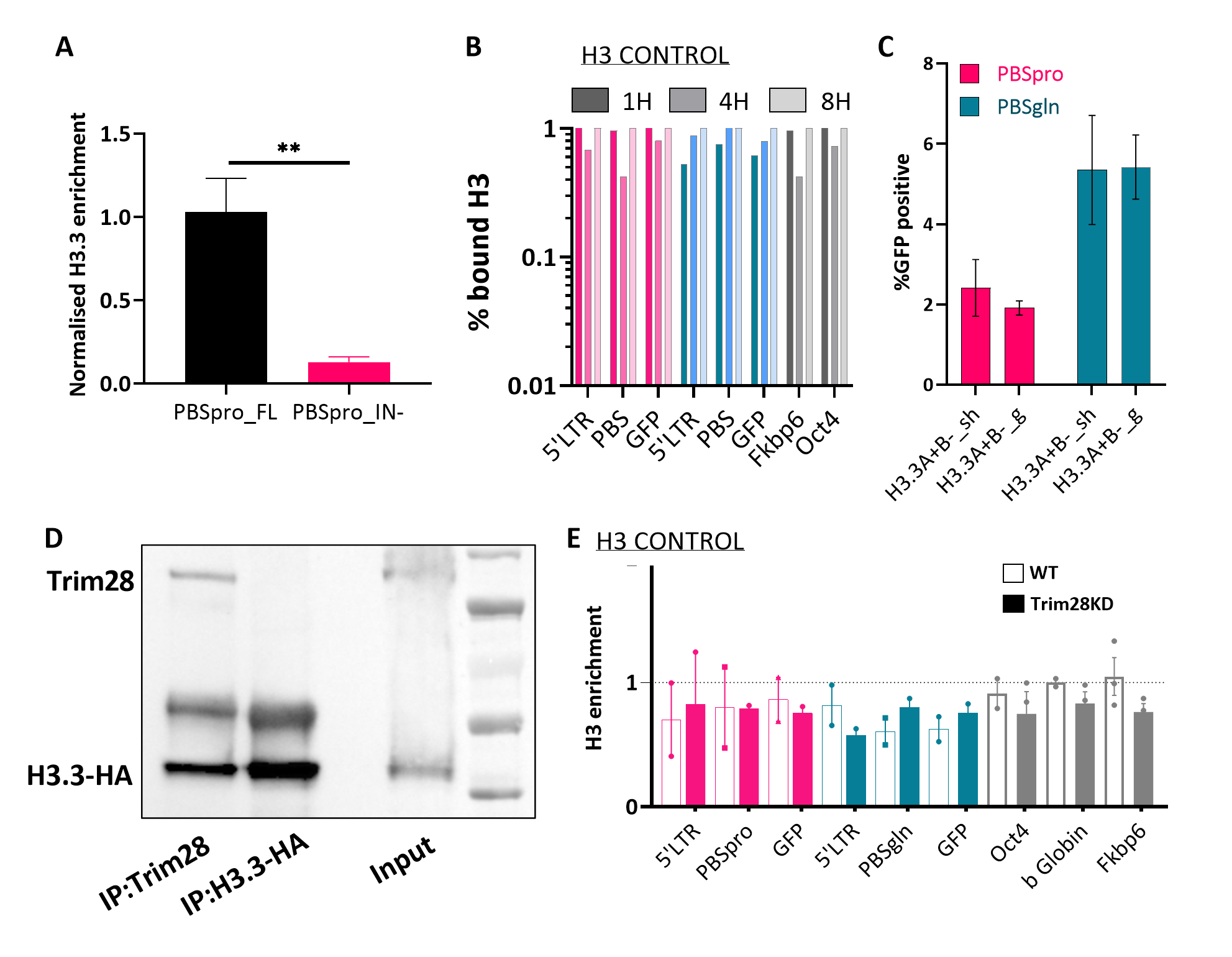

Supplement: Supplementary file 1 — Additional file 1: Figure S1. Controlling H3.3 deposition and dynamics.anti-HA ChIP on full length or integration deficient MLV-infected, Dox-induced KH2 cells followed by RT-qPCR on proviral site. Values are % Input normalized to the b-Globin gene. Data are the mean ± s.e.m..Accumulation of H3 into PBSpro, PBSgln, and genomic sequences at 1, 4 and 8 h after DOX induction. Presented are bound values normalized to the 8 h Bound fraction. ChIP-qPCR data are the mean ± s.e.m..Comparable levels of GFP positive cells in H3.3B KOand KDinfected with PBSpro or PBSgln MLVImmunoprecipitationof nuclear extracts from ESCs carrying the H3.3-HA-tag, followed by immunoblotting with the indicated Antibodies.ChIP-qPCR for H3 in cells depleted with WT and Trim28 infected with PBSpro or PBSgln virus. Data are the mean ± s.e.m.. [file 13148_2023_1499_MOESM1_ESM.tif]
